# ‘My story is like a magic wand’: a qualitative study of personal storytelling and activism to stop violence against women in Turkey

**DOI:** 10.1080/16549716.2021.1927331

**Published:** 2021-06-24

**Authors:** Kasonde Mwaba, Gamze Senyurek, Yeşim Işıl Ulman, Nicole Minckas, Peter Hughes, Sharli Paphitis, Shazana Andrabi, Lobna Ben Salem, Lida Ahmad, Ayesha Ahmad, Jenevieve Mannell

**Affiliations:** a Institute for Global Health, University College London, UK; b Amsterdam University Medical Centers, Vrije Universiteit Amsterdam, Department of Law, Ethics and Humanities, Amsterdam, Netherlands; c Department of History of Medicine and Ethics, Acibadem University School of Medicine, Istanbul, Turkey; d Springfield University Hospital, London, UK; e Institute of Psychiatry, Psychology and Neuroscience, King’s College London, London, UK; f Community Engagement Division, Rhodes University, Grahamstown, South Africa; g Centre for International Relations, Islamic University of Science and Technology, Awantipora, Jammu and Kashmir, India; h Department of English, University of Manouba, Tunis, Tunisia; i Independent consultant, Kabul, Afghanistan; j Institute for Medical and Biomedical Education, St George’s University of London, London, UK; k Department of Social Work, University of Johannesburg, Johannesburg, South Africa

**Keywords:** Maria Emmelin, Femicide, Turkey, survivors of violence, agency, gender-based violence

## Abstract

**Background**: Telling personal stories of violence has been central to recent advocacy efforts to prevent violence against women around the world. In this paper, we explore the use of personal storytelling as a form of activism to prevent femicide in Turkey. This study is part of a broader storytelling initiative called SHAER (Storytelling for Health: Acknowledgement, Expression and Recovery) to alleviate the psychological and emotional suffering of women who have experienced gender-based violence in high-prevalence settings.

**Objectives**: We conceptually explore personal stories of violence as a form of both distributed agency and activism. This conceptual framework is used to answer the following research question in the Turkish context: How do women use their personal stories of interpersonal violence for their own benefit (support) and that of others (activism)?

**Methods**: Our study is based on 20 in-depth semi-structured interviews with women who have experienced violence and were purposefully recruited by the ‘We Will End Femicide’ Platform in Istanbul. Interviews were conducted between March and August 2019. We used inductive and deductive thematic analysis to identify instances of personal storytelling at three levels: intrapersonal, relational and collective.

**Results**: Our results show how the use of personal storytelling can provide a means of healing from experiences of violence. However, this process is not linear and is often influenced by the surrounding context including: the listener of the story, their reaction, and what social networks the woman has to support her. In supportive social contexts, personal storytelling can be an effective support for activism against violence: personal stories can provide opportunities for individuals to shape broader discourses about violence against women and the right of women to share their stories.

**Conclusions**: Telling one’s personal story of violence can both support women’s agency and contribute to the collective struggle against violence against women more broadly.

## Background

Telling personal stories of interpersonal violence has been central to advocacy efforts in Turkey, as it has been throughout the world, with social movements such as *#metoo* changing the landscape of violence against women (VAW) activism and forcing widespread attention to women’s rights [1]. Personal stories of interpersonal violence are autobiographical stories and reflections about experiences of violence in interpersonal relationships, and often include stories of domestic violence, family violence, or child abuse. Personal storytelling can provide an opportunity for women to work through the trauma of experiencing interpersonal violence [2]. However, little is known about how women use their personal stories of violence, for what purposes, or why some women choose to tell their story as a means of acting against VAW more broadly.

In this paper, we explore how women in Turkey have used personal storytelling as a response to violence against women at an individual and societal level. Violence against women is common in Turkey with reported prevalence rates of intimate partner violence of between 13% and 78%, across different regions [3]. In recent years, feminist activists in Turkey have paid considerable attention to femicide [4] – the killing of women because they are women [5,6] – as one form of violence against women. While official statistics are largely unreliable on this issue, media reports account for hundreds of women being killed by their husbands or family members each year [6], and these rates appear to be increasing [7]. Despite this concerning trend, there is a dearth of research about femicide in Turkey and the women’s movement that has arisen in reaction to it.

To develop understandings of the role of personal storytelling in the development of VAW activism in Turkey, we conducted in-depth semi-structured interviews with 20 women who had experienced interpersonal violence and were affiliated with the ‘We Will End Femicide Platform’ in Istanbul between March and August 2019. Our analysis was guided by the following research question: How do women use their personal stories of interpersonal violence for their own benefit (support) and that of others (activism)? This article shares the results of these interviews, which highlight how personal storytelling benefits women at an intrapersonal level (e.g. reframing their own narrative of the violence), a relational level (e.g. sharing their story with others) and a collective level (contributing to broader social change through collective action). We then discuss what these results mean for the Turkish Women’s movement and anti-violence activism more broadly.

## Femicide and activism in Turkey

Over the past 40 years, the Women’s movement in Turkey has been instrumental in bringing about numerous changes to policies and laws concerning the place of women in Turkish society [8]. The organized resistance of women’s groups against domestic violence have helped increase the visibility of VAW as a social rather than private problem prompting action from the government and attention from academia and the media [8,9]. The women’s movement played a significant role in raising awareness and bringing visibility to the issue of VAW, getting the government to establish shelters for survivors of violence, the drafting of Law No. 4320 (as the first law addressing domestic violence in Turkey), and revisions to the Civil and Penal Code [10]. In 2014 Turkey became a signatory to the Istanbul Convention outlining comprehensive measures to prevent and combat VAW [11,12].

More recently, however, Turkey has experienced a backlash on gender equality and the status of women [13]. In July 2020, mass protests against VAW, organized in the wake of the brutal murder of student Pinar Gültekin, were violently cracked down on by police [14]. In early 2021, Turkey withdrew from the Istanbul Convention [15]. Moreover, the Turkish government and legal system have been complicit in openly expressing a patriarchal discourse of ‘proper’ female behavior and considering the provocation of men as a legitimate reason for violence [13,16]. This resurgence of traditionalism in Turkish society is underpinned by effective lobbying by Conservative groups who have argued that women’s rights, and the Istanbul Convention in particular, represent a threat to traditional Turkish family values [17].

In opposition to these recent changes, there has also been a rise of women’s and LGBT movements [13], including groups such as the ‘We Will End Femicide’ Platform, which regularly mobilizes women and organizes protests to bring awareness to the issue of VAW at the hands of men [11]. In reaction to the government’s withdrawal from the Istanbul Convention, there have been widespread protests by women across the country [18]. The telling of personal stories of interpersonal violence has played and continues to play an important role in collective action against femicide and VAW in Turkey. Recent social media campaigns have encouraged personal storytelling, including the hastag #*Sendeanlat* (‘share your story’), which encouraged women to share ‘their experiences of violence, intimidation and harassment,’ [19], and #*ChallengeAccepted*, which asked women to post black and white images of themselves to raise awareness of femicide and other forms of violence faced by Turkish women [17]. These campaigns highlight the importance of personal storytelling as a form of resistance to VAW in Turkey and the government’s inaction towards this issue.

Femicide and VAW are highly polarized issues in present day Turkey. On the one hand, Turkey is characterized by the infringement of women’s rights by the government and conservative groups, while on the other, the country is experiencing widespread protest and counter movements that are effectively using social media to create new spaces to challenge the broader normalization of violence [9]. This makes Turkey a highly interesting context to study the role of storytelling as a form of feminist resistance and activism against violence.

## Conceptual framework

In this paper, we approach personal storytelling as a dialogical interaction drawing on a social constructivist perspective [20]. This approach to personal storytelling lends itself to interpreting stories as both personal therapy [21,22], and a form of discursive activism [23,24]. Personal storytelling is understood as providing a space for recognizing and responding to private suffering [2], as well as a practice that can promote and enact women’s agency (i.e. their capacity to take action). It does this by offering opportunities for the creation of shared narratives through a collective and reciprocal telling of stories, with women acting as authors and protagonists of their own narratives [25,26]. The transformative potential of storytelling, therefore, is understood to exist for both the individual and the community.

This approach to personal storytelling as transformative in different spaces of women’s lives is aligned with Campbell and Mannell’s [27] notion of *distributed agency*. Agency, as one’s capacity to take action, is explored by Campbell and Mannell in relation to women’s ability to challenge or stop domestic violence against them (mostly by male partners). Drawing on symbolic interactionism, Campbell and Mannell [27] argue that the mediated capacity for women to act against violence is ‘distributed’ across time, space and social networks. From this perspective, women should not be defined exclusively as either ‘agents’ or ‘victims.’ Instead, a woman may be able to take actions that protect her from violent acts at certain times in her life (and not others), in certain spaces, and surrounded by certain people who facilitate her resistance. As suggested by Campbell and Mannell [24], activism sits at one end of a wide range of possible actions women can take to stop violence, which involves extending beyond one’s individual needs to address the broader social structures that drive violence in the first place.

In this article, we use the concept of distributed agency to explore the pathways and resources that support women who experience violence in Turkey to tell their personal stories of interpersonal violence for the purpose of broader social change. By focusing attention on women’s agency and activism, we are not discounting the importance of the significant structural barriers that both perpetuate VAW and undermine efforts to stop it. A focus on agency necessitates attention to these structural barriers and potential spaces of resistance [28]. As part of a focus on resistance, we are particularly interested in how social networks play a role in mediating women’s choices to use their personal stories as activism. This has important implications for both the social networks that may support women experiencing violence as well as those that support activists to address the broader social structures that drive VAW.

## Methods

### Study design

This qualitative study of women’s experiences of violence in Turkey was part of a broader research project investigating the potential for storytelling to alleviate the mental health impacts of VAW in women’s lives. The *Storytelling in Health: Acceptance, Expression, Recovery (SHAER)* project was initiated in 2018 with partners from Afghanistan, Kashmir (India), South Africa, Tunisia, Turkey and the UK to develop and pilot a storytelling intervention. This sub-study was part of an initial exploratory project to investigate the potential for the intervention to be developed for Turkey. Ethical approval was granted for the project by UCL (14,293/001) and Acibadem University’s Research Ethics Committee (ATADEK-2019/3).

### Sample

In-depth semi-structured interviews were completed with 20 women receiving support services and/or legal assistance from the ‘We Will End Femicide’ Platform (WWEF) in Turkey (Table 1). Participants were purposefully sampled from a list of survivors who had reached out to the Platform to seek help from violence with attention to obtaining a diversity of participants across different age groups and types of violence experienced. The age range of participants was between 18–49 years. Participants held a wide range of professional roles including: lawyers, nurses, shop assistants, private sector employees, clerks, market vendors, university students, and housewives. While most of the women had experienced intimate partner violence from a male partner, there were also instances of sexual violence and sexual harassment by non-partners.

**Table 1.** Participant details

Participant ID	Interview Date	Age	Place of birth	Education level	Type of violence
IDI1 (MO)	28/03/2019	34	İstanbul	01	Threat to right to life by stabbing (by husband)
IDI2 (ED)	15/04/2019	43	İstanbul	04	Threat to right to life by stabbing (by husband)
IDI3 (Gunes)	17/04/2019	49	Konya	04	Sexual harassment (by neighbour)
IDI4 (CA)	23/04/2019	30	İstanbul	05	Sexual harassment (by doctor)
IDI5 (Nur)	26/04/2019	38	İstanbul	03	Sexual abuse (father), stabbing (by husband)
IDI6 (MK)	06/05/2019	33	Bitlis	03	Dating violence
IDI7 (ED)	15/05/2019	32	İstanbul	05	Sexual harassment (by boss)
IDI8 (MB)	16/05/2019	32	Diyarbakır	05	Sexual abuse (by husband)
IDI9 (GU)	17/05/2019	35	İstanbul	05	Dating violence
IDI10 (BES)	22/05/2019	30	İstanbul	05	Sexual harassment (by peer student)
IDI11 (SA)	31/05/2019	27	İstanbul	05	Sexual abuse (by a stranger)
IDI12 (MG)	13/06/2019	18	Adıyaman	03	Sexual abuse (by teacher and cousin); ostracism (by family)
IDI13 (KT)	15/06/2019	31	Giresun	04	Dating violence
IDI14 (BT)	18/06/2019	21	Malatya	04	Sexual abuse (by father)
IDI15 (SB)	28/06/2019	24	İstanbul	02	Threat to life by stabbing (by husband)
IDI16 (EY)	28/06/2019	25	İstanbul	04	Dating violence, sexual harassment (by stranger)
IDI17 (ZY)	09/07/2019	41	Diyarbakır	01	Sexual abuse, armed assault (by husband)
IDI18 (EG)	23/07/2019	25	Ankara	05	Dating violence; sexual violence (by stranger)
IDI19 (HI)	27/07/2019	26	Rize	05	Dating violence
IDI20 (AB)	01/08/2019	33	Yozgat	01	Threat to life by armed assault (by husband)
Education Level: 01 Primary, 02 Middle school, 03 High school, 04 12th grade, 05 Higher education, 06 Vocational school					

### Recruitment

Women were first contacted by the WWEF Platform about the research study and asked if they would be willing to participate. Those who were willing to be interviewed were subsequently contacted by GS to arrange a secure and private location to meet and conduct the interview. The majority of interviews (13 of 20) were conducted at WWEF’s offices in the central district of Besiktas, Istanbul, but in cases where this was not possible because of the time and distance participants needed to travel, an office at Acibadem University School of Medicine (located in another part of Istanbul) was used instead. In one case, an interview was conducted in the participant’s home because of injuries resulting from the violence that made it impossible for her to travel. As a large metropolitan area, Istanbul boasts a diverse population from all areas of the country, and our sample reflects this diversity. Every effort was made to ensure that the interview location was secure and private for the participants to tell their stories.

Participants were given an information sheet and consent form at the beginning of the meeting. GS gave them time to read the information sheet and answered any questions the participants might have about the interview or study as a whole. Once the participants were comfortable with their involvement in the study they were asked to sign the consent form, which was kept securely by GS at Acibadem University’s premises.

### Data collection

The in-depth interviews were carefully designed and pilot tested to ensure a logical flow of questions that would minimize any potential distress for the participants in being asked to tell their stories of violence. Guidelines for interviewing women who had experienced violence were developed for the SHAER Project and shared with all members of the research team. Interviews were conducted by GS, who had extensive training and experience conducting sensitive interviews with vulnerable participants. The topic guide included questions about personal experience of violence, who they had told the story to and why, which was then followed by broader questions about why they thought violence against women existed, and what they thought could be done to address it. All interviews were audio recorded and transcribed into English by YIU in preparation for analysis.

### Data analysis

The data were analyzed using a combination of inductive and deductive thematic analysis [29]. As an initial step, two researchers (KM and JM) inductively coded a small selection of 3–4 transcripts each to identify initial concepts and themes, and inform the development of a clear research question that could be answered with the data following an emergent approach to qualitative research [30]. These themes and the potential direction of the analysis were then discussed with the project team and a plan for the analysis was established. Distributed agency was selected as our theoretical framework because of its ability to capture the non-linear and iterative process of women’s use of personal storytelling in response to experiences of interpersonal violence, and its empirical grounding in women’s experiences of violence in different settings globally [27,31].

Secondly, KM coded all the data deductively identifying any actions women had taken in response to experiences of violence and organizing these according to different dimensions of agency. As a third step, KM and JM discussed the organized themes and together developed a set of overarching themes that summarized the high-level contribution to the literature made by the study. Finally, KM returned to the data to cross-check these overarching themes and ensure they were robustly supported by women’s stories. The final overarching or global themes are summarized in our Results.

## Results

Participants described several actions they took in response to the violence they experienced. These are presented according to three dimensions of agency (intrapersonal, relational, and collective). Taken as a whole, the actions described by participants reveal agency as multifaceted with a range of different potential outcomes at intrapersonal, relational and collective levels, and highlight ways women use their personal stories of violence to achieve improved outcomes for themselves and others. Intrapersonal agency

Intrapersonal agency refers to individual changes in the thought processes of women regarding their experiences of violence, e.g. the realization that the violence they are experiencing is a problem. Participants clearly described the moment when their awareness changed to recognize the violence they were experiencing as a problem that required them to do something:

*I made up my mind to divorce him even to risk my own life. I could not put up with living like that. I was so determined. I set my mind to get divorced. I would rather die than live with a person I hate. (AB, age 33)*

Participants also described reaching different decisions about whether to speak out against the violence as it was happening or after it had happened. For example, Nur described standing up for herself against her abuser to avoid future episodes of violence:

*In the beginning of our marriage, he raised his hand against me, once. I shouted back at him, stop, I will break your arm! How on earth you dare to raise your arm against me? Who the hell are you to do this! He never did the same again. You get beaten either you shout back or stay silent. Shout at least and make everybody hear you. Perhaps someone hears me. I do not keep silent anymore. I am 38 years old. I will never remain silent. Not, anymore. I will never be a slave to any man. (Nur, age 38)*

Participants’ awareness of the violence as a problem often came about gradually, in response to the persistent use of violence by their attackers. For example, one participant described her numerous attempts to block her abuser from contacting her and her growing anger at the need to try and stop him:

*He kept on sending messages every day. I was not aware that he was posting about me via Facebook, as well. I had blocked him on WhatsApp. It occurred to me to check my Facebook account and I saw so many messages posted consecutively. I blocked him there, too. After this, things have gotten more out of control. He was opening new accounts 3-5 times a day and sending me messages from them over and over again. I blocked him, he kept messaging me. I closed my account eventually. I did not have to do this. I did not have to restrict my space. When you get angry you want to get rid of everything. Then he started to write on Twitter and Instagram. My Twitter account was not locked then. I locked them. I was changing my name constantly on Instagram. (BES, age 30)*

Other women described experiencing increased confidence to fight further violence in their lives. For instance, KT described how she first felt afraid but was now determined to not fall victim to violence again:

***Interviewer*** *Have you ever thought of litigating against him?*

***KT*** *: No. I was scared of him. But I am decided now, if he ever does something bad to me, harms me, I will surely file a suit. I have no fear from now on … . I will never surrender to a man again. I will never concede, not anymore. (age 31)*

Participants also described how their thinking had changed around women’s capacity to collectively challenge VAW in Turkish society and bring about change:

*What we do now, talking with each other, is a sort of women solidarity. Although it sounds like a slogan or cliché: if women get united they can create the world over again. This is not ‘manpower’. The age-old socio-cultural prejudices, motherhood problem, female problems, partner issues – all of them lay burdens on us all throughout history. We can stand up to them. I think, men cannot bear the menstruation pains we suffer even though we do not have masculine power. We are so powerful in the face of pressure and we go on living with resilience. All we need is to be conscious, aware of our problems. (MK, age 33)*

These shifts in perspective, or awareness-raising among participants, illustrates how women must first be conscious or aware that the violence they are experiencing is a problem requiring action before they are able to act. However, it is not always a straightforward process from awareness to action: participants described often going back and forth when deciding to leave relationships or taking further action against their abusers. Relational agency

Relational agency refers to the act of women informing someone about the violence they had experienced. This is ‘relational’ because it depends on the presence of another individual, however in many cases, telling someone about the violence was met by negative responses and didn’t actually stop the violence from happening.

Several participants described the negative experiences they had when telling their story of violence. As AB described, sometimes telling family members about the violence did not result in freedom from violence:

***Interviewer*** *How about your own family? How did you get in touch with them?*

***AB*** *: After that stabbing incident, when my body was black and blue all over, I wasn’t sent to a wedding party because my family would also be there. I managed to see them somehow and showed my body as it was, and I begged them to save me. My parents came to visit us to protest that beating incident. But my sisters in law held my mother and threw her down the stairs. My brother in law pulled out his weapon at my father. They told them that there was no return for me. We did not see each other for a long time after this. (AB, age 33)*

Other participants described situations in which telling their story of violence to others, including friends and family members, was met with hostility and judgment. In MK’s case, this reduced her capacity to tell others her story out of fear of how they would react:

***Interviewer*** *I see … Who did you tell this first? What were the reactions, then?*

***MK*** *: I first told this issue to one of my female friends. Her first comment was a masculine one. You married once. You have a daughter. Why did you need a boyfriend? She asked me. We had a quarrel. I was sorry to tell this to her. People get married, get divorced, they have the right to start over, I think. I did not talk about this later on. I was afraid of social prejudices. I could not share it with so many people. Sometimes I cried and cried all by myself. (MK, age 33)*

Negative reactions to women’s stories often involved the reiteration of traditional social norms as described by ZY when she shared her story with the abuser’s family:

***Interviewer*** *Whom did you first talk about him about what you had been through?*

***ZY*** *: Not so many people, not so much. I was talking with his sister in law. But she was giving me traditional advice that it would pass and that he was a man in the end and wives had to obey husbands, this and that. (ZY, age 41)*

Often police reactions to the violence also reiterated similar social norms about violence against women by intimate partners:

***Interviewer*** *I see, has your court case been concluded, yet?*

***EY*** *: The police were not so interested at first when they came. This case is seen as a private family matter, as an ex-boyfriend crisis. For instance, their attitude toward my second incident was so different from the previous one. The second case was an assault from someone that I didn’t know. In my first case police regarded the case as a family matter and did not attach so much importance to it. They said there was no violence: ‘it was like the physical expression of feeling towards you’, this is what they said. (EY, age 24)*

In contrast, some participants described how sharing personal stories of violence with another person had the ability to inspire others to open up about their own experiences:

***ED*** *I hope people tell stories at friendly circles. That is a story for you. It is as if my story is a magic wand. Perhaps you touch somebody's life and make her open up..*

In Nur’s case, the sharing of a story of violence by her sister was the event that inspired her to take action against her father and report the violence to the police

*She told me that our father raped her. I felt as if I was crushed under that 5-story block of flats because I was feeling like a mother. I was terrified. She told me that he kept raping her for a long time. I lost my temper then. Everything she told was so familiar to me. It was the same that I had experienced. I did not think that he would do the same thing to his own biological daughter. I asked how long he had been doing this. She said that she had been raped since she was 9 years old. She even got abortion from her own biological father. I was furious … Then I told my own story to my sister. I said, ‘it is time to make an official complaint at the court, are you with me?’ She replied ‘I am with you, of course I am. What can we do’? I said ‘Come on, we are going to the police station right now. (Nur, age 38)*

Positive responses to women’s stories were often achieved when participants were able to access knowledgeable or influential people they knew for advice and assistance. For instance, SA is a lawyer who was able to draw on her personal contacts as a means of instigating a legal response to her attempted rape:

*I called a person with a vast network whom I have known since my clerkship years, just after the incident. I told him about the situation and asked for his help. I did this because the police are rather ineffective, they don’t take you seriously. They underestimate your situation. I remember a girl that had been raped – the police asked for sperm samples from her and she was told by the police that the samples were sent to Ankara and they did not know when the results would come. They make things more difficult, so I kept pressuring them. This is the reason I asked for help from that influential person. (SA, age 27)*

Another participant, Gunes, was able to use a report to the police as a means of convincing her husband to stop the violence:

*One day he beat me. I called the police station. I told them that my husband was beating me. They sent a team of policemen. Then he left the house, but after a while he came back. We made peace. I told him that I would call the police again if he did the same thing. He said he would not do it again. He never attempted to beat me again from that moment on. (Gunes, age 49)*

Women told their stories to a range of different individuals including friends, family, the police, and the WWEF Platform. When these reactions were negative, women often stopped telling others or hesitated to do so. When reactions were positive, the violence often stopped, women were able to leave violent relationships, and court cases moved forward.

### Collective agency

Collective agency was defined as actions taken in relation to bringing about wider change or challenging the status quo regarding VAW, e.g. taking part in marches. Participants described making media appearances, either in newspapers or on television shows to raise awareness of their plight and issues of VAW in Turkish society. For instance, MK spoke about how the WWEF Platform helped her make her case to the Turkish Parliament:

*We were able to reach members of the Parliament. Two members of the Turkish National Assembly brought my case to the agenda of the Parliament by submitting parliamentary petitions. Franky speaking, however, our question was never officially replied to. But this only happened after I consulted the Platform. (MK, age 33)*

Other participants not only described the actions that they had taken to try and bring about change, but also described how this was a means of having their voices heard and encouraging other women who may be facing similar situations:

*I would like to speak out. I am keeping cool indeed, at the Police Station, at the General Prosecutor’s Office. I was on a women’s programme on TV. I would like to empower other women. I can raise hope for people if I have a voice myself. (GU, age 35)*

Participants described being active in women’s platforms, like WWEF Platform to raise awareness about VAW and push for change. They described volunteering with women’s platforms, taking part in marches and appearing at court cases to support other victims of violence. These actions all belonged to an interest in working collectively with other women to bring about change:

*This made me think that we are all at stake. Not only me, but all the women are potentially in danger, I am concerned with all women, not only myself. As I am a lawyer, a sensitive citizen, I wanted to be beneficial for all women. This is not something personal. The reason for my initiative was not only this incident, I was thinking of doing something to the benefit of women at an NGO, even while I was studying at the university. However, you think that you will do it some day, and you keep on postponing to start actually. Now the day has come. Following this attack, I focused on how to help women. (SA, age 27)*

Many participants described experiences similar to SA’s, e.g. sharing their experiences of violence in media appearances and engaging in activities with NGOs supporting women’s rights and fighting VAW. In participating in these broader advocacy activities, they show concern for others as they hope to go beyond themselves and bring about change on a wider scale in society, e.g. changing social norms.

## Discussion

This study aimed to address gaps in the literature surrounding the personal storytelling experiences of women who had experienced interpersonal violence. We have explored the role that telling their personal stories of violence has played in women’s efforts to improve outcomes for themselves and others, as an example of their agency in the face of violence. At an intrapersonal level, women described specific moments of awareness or change in the way they perceived their individual story of violence. This often involved moving away from an acceptance or resignation of the violence towards a recognition of the need to act in order for the violence to stop (e.g. leaving their husband, asking others for help, etc.). These examples of intrapersonal agency also happened at different levels: some women described becoming aware of the need to change their immediate situations while others described an awareness of the broader social norms that condone violence, which highlights the different reasons why women choose to tell their personal stories of interpersonal violence.

At a relational level, women described their experiences of telling others their story of violence, often as a means of seeking help. Many women experienced negative reactions to their efforts to tell their stories to others, which highlights the persistent social norms that condone VAW in Turkish society. These negative reactions to women’s stories often stopped them from taking further action or seeking help from others. In contrast, when reactions to women’s stories were positive and supportive, this often started a sequence of events whereby the violence stopped and perpetrators were brought to justice. Positive reactions from others has also been shown to have significantly positive mental health affects for women experiencing violence [2]. While telling a friend a personal story of violence may not be as effective as physically leaving a violent partner, it can have an important effect on alleviating the feelings of isolation and self-blame that often accompany experiences of violence [32,33].

At a collective level, women described using their personal stories as part of a broader movement for social change. This included their efforts to tell their stories to a broader public audience, including through social and traditional forms of media. Women described the importance of storytelling about interpersonal violence as part of a wider narrative of women’s rights and activism against violence. Several women described volunteering with women’s platforms, engaging in marches and using social media to raise awareness of VAW. Such acts of collective agency on the part of women’s groups and networks have historically played an important role in bringing attention to issues of VAW both domestically and internationally [34]. Moreover, as Zeynep Gulru Goker argues, storytelling itself can provide a form of collective action as personal stories of interpersonal violence increasingly become recognized as part of the public sphere [35].

Drawing on the concept of *distributed* agency in our analysis highlights how the three dimensions of agency vary between women and the contexts in which they live, e.g. time, space and social networks [27]. There were stark variations in the amount of time women needed to process their stories of violence at an intrapersonal level and become aware of the need for them to take action in order to change the situation. While the majority of women described how their awareness of the situation came about gradually, for a few women their awareness happened almost instantaneously due to their personal beliefs or previous convictions concerning VAW. This is consistent with the mental health literature on VAW, which suggests that the controlling behaviours and psychological violence that often coincide with physical violence play an enormous role in undermining women’s ability to perceive the violence as not their fault and consciously decide to change their circumstances [36]. It also highlights that while some women affected by violence may already hold beliefs that lend themselves to activism, others may become activists through processing their own experiences of violence.

Lastly, social networks played a key role in supporting women’s agency in our study, particularly in the responses women received when telling their stories to others. Women with the social capital to circumvent unhelpful police officers and call on powerful allies were more successful in having their cases heard in court than those who did not have this social capital. This is consistent with findings from other studies about the intersections between social capital and VAW [37], and is especially important to consider in contexts where the agency of women is limited as a result of their social marginalization [38].

These results contribute to a growing body of literature on the distributed nature of women’s agency in response to violence globally. The dimensions of agency used to categorize our results correspond with other similar frameworks, including those used to understand women’s experience of violence in Rwanda [39], and VAW among people living with HIV in Haiti [31]. Other studies also highlight the importance of exploring agency as a contextual phenomenon. For instance, a study of young women living in informal settlements in South Africa [40] highlights how many women often choose to stay with violent partners because they feel loved and respected, and that the violence is sometimes a relatively minor challenge in otherwise difficult and often precarious lives. This emphasizes the need for further research into how personal stories of interpersonal violence contribute to activism in other contexts with different socio-political dynamics than those currently experienced in Turkey.

As mentioned, hashtags and social media posts that encourage the telling of personal stories of violence has been a key strategy of the anti-femicide social movement in Turkey [17,19,23]. Our findings contribute to this literature by highlighting women’s perspectives on the value that participating in these public online debates can have. Anonymous hashtags and social media campaigns may play an important role in helping women circumvent the social structures that often silence women in the face of interpersonal violence [39]. However, in cases where women are unwilling or unable to tell their stories of interpersonal violence to family, friends or the police because of the negative reactions they may receive from both formal and informal sources of support, the anonymous sharing of these personal stories can offer an alternative means of sharing personal experiences as well as a form of radical politics [35]. In this way, social media campaigns can provide a space for women to tell their personal stories as part of a broader social movement when those close to them are unable to hear their stories with compassion.

### Limitations

As a study about women’s personal stories of violence, men’s stories about violence are not accounted for, which obscures stories of how men are also involved in activism to prevent VAW. In addition, as participants were selected from a group of women who had approached the WWEF Platform, the stories of violence and activism included in this paper are not representative of the Turkish population as a whole and instead represent a select group of women who have been able to draw on social structures and discourses that enable them to tell their own personal stories, consistent with the aims of qualitative inquiry. Since women included in this study were recruited through an online anti-femicide platform, our results do not easily reflect engagement with non-digital spaces, however, they do highlight the continued importance of digital spaces in supporting women’s activism against violence in Turkey. The study design did not lend itself to drawing conclusions about the impact of personal storytelling on either women’s physical or psychological health outcomes, women’s future experiences of violence, or the effectiveness of participation in anti-violence campaigning on women’s lives, and this would be a rich area for further research.

## Conclusion

Telling one’s story of violence can be a powerful support for the agency of women who have experienced violence in Turkey. However, a distributed approach to agency also helps acknowledge the complexities of how storytelling plays out both in the lives of women storytellers and in broader spaces for social change through activism. Personal storytelling can bring about broader social change if the components of time, space and social networks are in place for the story to be compassionately received and acted upon [2]. Women who experience violence and choose to report it are often required to repeat their story of abuse to multiple others (e.g. friends, family, police, lawyers, courts of law, etc.). This storytelling may support both the woman’s mental health and physical wellbeing (e.g. if they tell a friend who then provides shelter), but it can also lead to additional acts of violence (e.g. being mistreated by police after reporting rape). The role of personal storytelling in developing activists against VAW therefore needs to be taken with a balance of caution. For some women, personal storytelling plays an important role in their personal awareness of the situation, the actions they are able to take to address it, and their capacity to work collectively to transform social norms more broadly. This represents the role personal storytelling can play in harnessing agency and shaping activism in the face of violence. However, it is also likely the exception – women who use their personal story as a form of activism are surrounded by people and contexts that make this possible.

While it is important to acknowledge the structural constraints that may limit the reduction of violence achieved through personal storytelling, this should not obscure the critical importance of the telling of women’s stories as a social process. Even if personal storytelling does not achieve help for the woman’s individual situation, the sacrifice women have made through telling their story is still worth more because the story is told. Women who tell their stories against a background of social and structural constraints are taking enormous psychological and physical risks. Personal storytelling about experiences of interpersonal violence in a social and cultural landscape that undermines women’s human rights can instigate additional acts of violence and sometimes even death. The motivation for taking such risks may be help-seeking, but it can also be something much larger than this – women tell their personal stories of violence because they hope their story will be shared with other women who are then able to achieve a different ending.

## Supplementary Material

Supplemental Material

## Endnote: A collaborative definition of personal storytelling

This article was written collaboratively by a multidisciplinary group of academic activists specializing in global health, political science, literary studies, philosophy, psychology and psychiatry. We come from places such as Turkey, Afghanistan, Tunisia, South Africa, the UK and Kashmir. As part of the SHAER (Storytelling in Health: Acceptance, Expression, Recovery) project, we have defined our own conceptual understanding of personal storytelling as any first-person account in which a person explores their own ways of being, perceiving, and understanding themselves in their life-world. The process of sharing a personal story is understood as a dialogical interaction that plays an important role in human and moral development, allowing both the storyteller and the listener to come to a deeper understanding of themselves, the contexts in which they are embedded, and their interconnectedness. The significance of a personal story is therefore to be found in the shared experience through which it is both given and received. However, the ideal conditions for this dialogical interaction are not present in all contexts, most typically in situations where power hierarchies and epistemic injustice rupture the relationship between speakers and listeners, which is the case in many of our countries. When oppression and credibility deficits characterize the dialogical interactions in society, members of oppressed or marginalized groups may turn to activism in order to force the hearer to listen to them.

## Paper context

All data was collected in Turkey and analysed by an international team of collaborators as part of the SHAER Project (www.shaerstorytelling.org)
