# Supplementary material for: ‘My story is like a magic wand’: a qualitative study of personal storytelling and activism to stop violence against women in Turkey
Source: Glob Health Action. 2021 Jun 24;14(1):1927331. doi: 10.1080/16549716.2021.1927331 (PMC8231392; doi:10.1080/16549716.2021.1927331)
Supplement: Supplemental Material [file ZGHA_A_1927331_SM7011.docx]

**Acknowledgments**: We are grateful for the involvement of the WWEF Platform for making this study possible, and in particular Ms. Gulsum Onal, M.D. and Ms Aysen Ece Kavas who were instrumental in contacting potential participants. We would also like to sincerely thank the women who participated in the study and agreed to tell us their personal stories.

**Author contributions**: JM conceptualized the paper and the first draft was written collaboratively by KM and JM. GS and YU did the data collection in Turkey with input from NM and JM on methods and ethical guidelines. AA, GS, YU, LBS, SP all provided original written content for the introduction, conceptual framework and discussion. JM, AA, YU, LBS, LA, SA, SP, PH are investigators on the SHAER study to explore the potential for storytelling to alleviate the mental health impacts of violence against women in high-prevalence settings, funded by the UK’s Medical Research Council and Art and Humanities Research Council. This project was a multi-disciplinary collaboration of scholars from psychology, psychiatry, political science, philosophy, bioethics and literary theory. This paper reflects the diversity of our disciplinary perspectives. All authors contributed to editing and approving the final paper.

**Disclosure statement**: We have no conflicts of interest to declare.

**Ethics and consent**: Ethical approval for the study was granted by UCL’s Ethics Committee (14293.001) and Acibadem University in Turkey (ATADEK-2019/3)

**Funding information**: Support provided by the UK’s Medical Research Council and Art and Humanities Research Council

**Paper context**: All data was collected in Turkey and analysed by an international team of collaborators as part of the SHAER Project (www. shaercircle.com)
